# Hormone replacement therapy and mammographic density: a systematic literature review

**DOI:** 10.1007/s10549-020-05744-w

**Published:** 2020-06-22

**Authors:** Shadi Azam, Katja Kemp Jacobsen, Arja R. Aro, Elsebeth Lynge, Zorana Jovanovic Andersen

**Affiliations:** 1 Unit for Health Promotion, Department of Public Health, University of Southern Denmark, Niels Bohrs Vej 9, 6700 Esbjerg, Denmark; 2 Department of Technology, Faculty of Health and Technology, University College Copenhagen, Copenhagen, Denmark; 3 Section of Environmental Health, Department of Public Health, University of Copenhagen, Copenhagen, Denmark

**Keywords:** Mammographic density, Hormone replacement therapy, Breast cancer risk, Systematic literature review

## Abstract

**Purpose:**

Hormone replacement therapy (HRT) is used to reduce climacteric symptoms of menopause and prevent osteoporosis; however, it increases risk of breast cancer. Mammographic density (MD) is also a strong risk factor for breast cancer. We conducted this review to investigate the association between HRT use and MD and to assess the effect of different HRT regimens on MD.

**Methods:**

Two of authors examined articles published between 2002 and 2019 from PubMed, Embase, and OVID using Covidence systematic review platform. Any disagreements were discussed until consensus was reached. The protocol used in this review was created in accordance with the Preferred Reporting Items for Systematic Reviews and Meta-Analyses (PRISMA). Quality of each eligible study was assessed using the Oxford Center for Evidence-Based Medicine (OCEBM) hierarchy.

**Results:**

Twenty-two studies met the inclusion criteria. Six studies showed that using estrogen plus progestin (E + P) HRT was associated with higher MD than estrogen alone. Four studies reported that continuous estrogen plus progestin (CEP) users had higher MD than sequential estrogen plus progestin (SEP) and estrogen alone users. However, two studies showed that SEP users had slightly higher MD than CEP users and estrogen alone users.

**Conclusions:**

Epidemiological evidence is rather consistent suggesting that there is a positive association between HRT use and MD with the highest increase in MD among current users, and CEP users. Our results suggest that due to increase in MD and masking effect, current E + P users may require additional screening procedures, shorter screening intervals, or using advanced imaging techniques.

**Electronic supplementary material:**

The online version of this article (10.1007/s10549-020-05744-w) contains supplementary material, which is available to authorized users.

## Introduction

Hormone replacement therapy (HRT) is recognized as an effective treatment for alleviating the climacteric symptoms of menopause such as hot flushes, sleeping disturbance, depressive mood, muscle and joint pain [1]. Large clinical trials showed that use of HRT prevents bone loss and decreases risk of osteoporosis and bone fractures in menopausal women [2, 3]. The most common HRT regimens are: estrogen alone, combined estrogen plus progestin (E + P) either as continuous estrogen plus progestin (CEP) or sequential estrogen plus progestin (SEP) [4]. For many years, HRT was used widely to improve the quality of life of menopausal women. However, after the results from two large population-based studies, the Women’s Health Initiative (WHI) study in the United States in 2003 [5] and Million Women Study (MWS) in the Unites Kingdom in 2003 [6] showing that HRT use increases risk of breast cancer and cardiovascular disease, the long-term benefits and potential adverse effects of HRT on menopausal women were reconsidered. After the results from these two studies, new guidelines concerning amount, types, and duration of HRT for menopausal women were released, leading to a decline in use of HRT along with a reduction in the rates of breast cancer incidence by 6.7% among American women. After 2003, dramatic decline in HRT consumption and breast cancer incidence were recognized as the consequence of the findings from WHI and MWS studies [7].

Mammographic density (MD) is a very strong predictor for breast cancer risk [8]. MD refers to the amount of radiologically dense breast consisting of epithelial or stromal tissue that appears light on a mammogram, whereas fat tissue appears dark on a mammogram [9]. There are different methods for measuring MD including percent mammographic density (PMD), Breast Imaging Reporting and Data System (BI-RADS), and Wolfe [10]. Women with very dense breasts (> 75% density in the breast) have a four to six times greater risk of breast cancer than women with little density (< 5% density) or fatty breasts [8, 9]. Previous studies suggested that MD is influenced by several exogenous hormones which are known to influence breast cancer risk thus, MD is known an important surrogate marker for the effects of exogenous hormones on the risk of breast cancer [11, 12]. This review used a systematic approach to explore the association between HRT use and MD. Furthermore, we investigated the effect of different HRT exposure states (never, former, current use) as well as different HRT regimens on MD.

## Methods

### Inclusion and exclusion criteria

Studies included in this review met the following criteria: original research in peer-reviewed journals, full-text available online, a randomized controlled trial (RCT) as the study design, a cohort (prospective-cohort or retrospective cohort), a case–control, or a cross-sectional with a clear description of the samples and methodology, and articles available in English language. We focused on studies that examined the associations between HRT use and MD. Additionally, the MD assessment criteria in the studies had to be based on either Wolfe, PMD, or BI-RADS category. We excluded any descriptive manuscripts which did not have the focus on the association between HRT and MD.

### Search strategy

Epidemiological studies from July 2002 to 2019 were retrieved from the following databases: PubMed Central (US National Institutes of Health [NIH]), OVID, and Embase using the following combinations of MeSH terms: “hormone replacement therapy”, “postmenopausal hormone replacement therapy”, “estrogen-progestin hormone replacement therapy”, “combined hormone replacement therapy”, “HRT”, and “estrogen alone hormone replacement therapy” in conjugation with “mammographic density” and “breast density”. An example of the search strategy used in one of the search databases (PubMed) is found in "Appendix".

### Study screening

We imported and managed all study citations identified from the search strategy using the Covidence systematic review platform [13]. Two reviewers (S.A, K.J) independently screened the titles, abstract and reviewed the bibliography of articles found through electronic search engines for eligibility. Two pairs of co-authors reviewed all the abstracts. Disagreements occurred in less than 5% of all articles; any disagreements were discussed until consensus was reached.

### Review protocol

The protocol used in this systematic review was created in accordance with the Preferred Reporting Items for Systematic reviews and Meta-Analyses 2009 (PRISMA) statement and flowchart was used [14]. Supplementary Table 1 illustrates the PRISMA checklist of this systematic review. Furthermore, in this literature review, quality of each eligible study was assessed using the Oxford Centre for Evidence-based Medicine – Levels of Evidence (OCEBM) hierarchy [15]. The OCEBM levels is a widely used system, which categories studies into different levels ranging from 1 to 5 based on their study designs; it helps the researcher to evaluate the reported results. In The OCEBM system, levels 1, 2, 3, 4 and 5 represent well-designed and high quality RCTs, prospective and retrospective cohort studies, case–control studies, cross-sectional and case-series studies, and expert opinion or unpublished clinical observations, respectively [15]. Level 1 is the highest quality and level 5 is the lowest [15]

**Table 1:** Characteristics of epidemiological studies investigating HRT use and mammographic density

Author, year	Country	Study design	Quality of study design	Sample size and characteristics	HRT regimens	MD assessment	Confounders included in the final analysis	Results/Findings
Byrne et al. (2017) [18]	United States	Case–control	3	- Cases: 174 women who developed breast cancer - Controls: 733 healthy women - Age range 50–79 years - MD was assessed from mammograms taken prior and one year after randomization of cases and controls	CEP	–	- Age - Baseline BMI - Clinical center - Age at first birth - Parity	- Women assigned to CEP group had a larger and broad distribution of mammographic density change (mean change = 9.7%), whereas women in never HRT users/placebo group exhibited minimal mammographic density change over one year (mean change = − 0.05%) - After adjusting for covariates including baseline density, the difference in mean change in mammographic density between the placebo (− 0.65%, 95% CI = − 1.86 to 0.55) and the CEP users (9.49%, 95% CI = 8.25 to 10.72) treatment arms was statistically significant (*p* < .001)
Olsson et al. (2014) [33]	Sweden	Cohort	2	- 619 women with incident breast cancer - Age rage 48–81 years	–	BI-RADS	–	- From 214 women with dense breast 106 (49.5%) were HRT users at the time of breast cancer diagnose and 46 (21.5%) were never HRT user
Carmona-Sanchez et al. (2013) [29]	Spain	Cohort	2	- 165 postmenopausal women - 1-year study follow-up	- Estrogen alone - CEP - SEP	BI-RADS	–	- MD increased in 7.9% of women receiving estrogen alone compared to 25.2% women receiving CEP (*p* < 0.022) during 1 year. - After 5 years of HRT 7.9% of women versus 28.3% of women (*p* < 0.009) had MD increase, respectively - There was significant statistical difference in women treated with estrogen alone versus those treated with combined HRT - After 5 years of HRT, MD increased 21.8% in women receiving SEP versus 38.8% in those under CEP (*p* < 0.039)
Crandall et al. (2012) [20]	United States	RCT	1	- 695 postmenopausal women - Age range 50–79 years - 1-year follow-up study	- Estrogen - E + P	PMD	- Age - Ethnicity (Caucasian, Black, American Indian, Asian Pacific Islander, unknown) - BMI (kg/m^2^, continuous and quartiles) - Gail risk score	- At 1-year follow-up the change from baseline in PMD was 1.4% for estrogen alone users and -0.8% for never HRT users - The PMD change for E + P users was 6.3% and for never HRT users was − 0.9% - Changes in PMD were statistically significantly greater among women assigned to active therapy than among women assigned to placebo and were more marked among women assigned to E + P than estrogen alone - The result from this study cannot be assumed to apply to other types, doses, routs of estrogen or progestin therapy
Couto et al. (2012) [19]	Norway	Cross-section	4	- 2424 postmenopausal women - Aged 50–69 years	- Estrogen - E + P	PMD	- Age at screening - BMI - Number of children - Age at first childbirth - First-degree family history of breast cancer - Number of years spent in school	- PMD was higher (19.6% with 95% CI, 18.3–20.8%) in ever users of HRT compared to never users (16.3 with 95% CI, 15.7–16.8%) - The highest PMD was found in current HRT users (22.6% with 95% CI, 22.1–23.2%), followed by former users (17.7% with 95% CI, 17.2–18.2) and never users (16.3% with 95% CI, 15.7–16.8%) - Current E + P users had a significantly higher PMD 25.4% (24.6–26.1%) than current estrogen users 18.9% (17.6–20.2%) and never HRT users 16.3% (15.7–16.8%) - In this study, MD was measured only once and relied on cross-sectional mean differences between the study groups, rather than changes in density following start of HRT use
Yaghjyan et al. (2012) [35]	United States	Nested Case–control	3	- 522 premenopausal women - 599 postmenopausal women - Age ≥ 40 years - Cases: 265 women with high MD - Controls: 860 women low MD	–	BI-RADS	- Parity - Age at first - Child’s birth were modeled as categorical with three levels (parity 0, 1 –2, ≥ 3 age at first child’s birth < 20, 20 –29, ≥ 30)	- Postmenopausal women with history of HRT use had increased odds of higher MD (OR 2.1; 95% CI 1.4–3.3) and compared to postmenopausal women who never used HRT - In this study, due to the lack of racial heterogeneity, 99% were White-non-Hispanic and therefore the findings are limited to one racial group
Jeon et al. (2011) [32]	Korea	Cross-section	4	- 516 women with age range between 40–80 years - 284 premenopausal women - 232 postmenopausal	–	BI-RADS	–	- Use of HRT was positively related to higher MD, the odds of having dense breasts increased by OR = 2.13 (95% CI; 1.09–4.16) for women who used HRT compared to never HRT users
Boyd et al (2011) [17]	Canada	Case–control	3	- Case: 1164 women with breast cancer - Control: 1155 women - Age 40 to 70 years	–	PMD	- Age, BMI, Age at menarche - Parity (parous or nonparous), Number of live births - Age at first birth - Age at menopause (except in analysis of premenopausal women) - Breast cancer in first-degree relatives (none, one, two)	- Among cases PMD was greater in current than in never HRT users (difference, 6%; *p* < 0.001) and greater in past users than in never users (difference 3.4%; *p* = 0.03) - Among controls current use of HRT was associated with a slightly greater mean PMD (difference, 1.6%; *p* = 0.26) than in never users, and past users of HRT had lower PMD than never users (difference 3.8%; *p *= 0.01)
Chen et al. (2010) [30]	Taiwan	Cohort	2	- 467 postmenopausal women - Age 43–69 years	- Estrogen - E + P	BI-RADS	- BMI - Age at menopause - Age at start of HRT - Duration from onset of menopause to the start of HRT	- The duration of HRT use was positively associated with increase in MD (*p* < 0.001) - Women using E + P, the probability of increased MD was progressively increased as the duration of administration extended (from 7.5% to 22.4%) but not in women who used estrogen alone - Women using E + P for more than 4 years had significant increase in their mean density score, compare with those using only estrogen alone (*p*= 0.013). However, after adjustment for effects of other variables, the association between choices of HRT regimens (E + P vs. estrogen alone) did not reach the significance level
Crandall et al. (2008) [21]	United States	RCT	1	- 428 Postmenopausal women - Age range 45–64 years	- Estrogen alone - CEP - SEP	PMD	- Baseline mammographic density - Age - BMI - Change in BMI (12 months minus baseline) - Daily alcohol intake - Parity (none versus 1–2 versus ≥ 3 pregnancies) - Cigarette smoking (current versus not current) - Ethnicity (Caucasian versus not Caucasian) - Physical activity - Age at first pregnancy	- The mean serum estrogen sulfate (E1S) level changed after 12 months compare to baseline level was 1.93 ng/mL for estrogen alone users, and the difference was more pronounced in E + P users 2.49 ng/mL (*p* =0.02) - Change in E1S level and change in MD after 12 months were significantly positively correlated (*p* = 0.0001). This is for every 1 nmol/L increase in E1S level at 12 months follow-up, PMD was 1.3% higher. E1S-MD association was more pounced in women taking SEP compare to estrogen alone users The increase in PMD was 0.5% (SD, 5.1%) among never HRT users, 1.2% (SD, 7.5%) among estrogen alone users, 4.9% (SD, 8.7%) among SEP users, and 4.7% (SD, 10.8%) among CEP users Change in PMD was significantly more pronounced among E + P users than estrogen alone users
Harvey et al. (2008) [24]	United States	Case–control		- Case: 28 postmenopausal women using HRT - Controls: matched with 28 postmenopausal women not using HT at the time of breast cancer diagnosis - Age range: 45–84 years		PMD	- Age - HRT status	- There was a statistically significant difference in breast density noted between the HRT users and never HRT users groups (*p* < 0.0001) with a median difference in MD 54% for HRT users and 31% for non-HRT users - Percent fibrous stoma was 7% higher for HRT users compared with non-HRT user; however, the difference was not statistically significant. - Increasing MD in women using HRT was associated with increased fibrous stroma (*p* = 0.02)
Duijnhaven et al. (2007) [27]	Nederland and UK	Cohort	2	From Netherland - 620 HRT users - 620 never HRT users with - Age range between 49–69 years From UK - 175 HRT users - 161 never HRT users - Age 51 to 71 years	- Estrogen - E + P	PMD	- Type of HRT use (no HRT use, ET use, combined HRT use, or tibolone use) - Density at first mammogram - Age, BMI - Age at menarche - Parity/age at first full term pregnancy (nulliparous, ≤ 25 years, and ≥ 25 years) - Menopausal status (Premenopausal, perimenopause/postmenopausal) - Family history of breast cancer - Previous oral contraceptive use - Smoking (0, < 5, 5–15 and ≥ 15 pack-years) - Alcohol consumption - Physical activity (inactive, moderately inactive, moderately active, active) - Study population (Prospect-EPIC/EPIC-Norfolk)	- PMD at the first mammogram was lower for never HRT users (37.0%) than for estrogen alone users (39.3%) and E + P (46.1%). The dense area at first mammogram was lower for never HRT users (40.6 cm^2^) than for estrogen alone users (45.9 cm^2^) and E + P users (50.8 cm^2^) - At the second mammogram the absolute mean density was lower for never HRT users (31.7%) than for estrogen alone users (32.6%) and E + P (35.6%). The dense area at the second mammograms was lower for never HRT users (38.08 cm^2^) than estrogen alone users (40.78 cm^2^) and E + P users (41.93 cm2). The median between the first and second mammogram was 3 years - Longer use of HRT (> 1 year) appeared to have a larger effect on MD than shorter use of HRT (< 1 year)
Aiello et al. (2006) [28]	United States	Cross-section	4	- 39,296 postmenopausal - Age ≥ 40 years	- Estrogen - E + P	BI-RADS	- Age at mammogram - BMI - Age at first birth (5-year intervals) - Type of menopause (natural, bilateral oophorectomy with or without hysterectomy, hysterectomy only, hysterectomy with unknown oophorectomy, and other)	- The Odds of having dense breast increased significantly in current HRT users by OR 1.91 (95% CI, 1.81–2.00) and for former HRT users increased by 1.14 (95% CI, 1.08–1.21) compared to never HRT users - Current E + P users had significant increase in odds of having dense breasts (OR 1.98; 95% CI 1.87–2.09) and estrogen alone users had significant increase in the odds of having dense breasts by (OR 1.71; 95% CI 1.56–1.87) compared to never HRT users - In this study, the study population is largely white, which may limit the generalizability of the results to other race
Crandall et al. (2006) [22]	United States	RCT	1	- 875 postmenopausal women - Aged 45 to 64 years - 1-year follow-up study	- Estrogen - CEP - SEP	PMD	- Age - Parity - Age at first pregnancy - BMI - Alcohol intake - Smoking - Ethnicity - Baseline PD - Treatment assignment (Placebo, conjugated equine estrogens, or progestin-containing regimen)	- At 12 months mean PMD had significantly increased in SEP and CEP users by 4.6% and 4.4%, respectively - The change in PMD was 4.0% in the progestin-containing arms, and it was significantly higher than that in estrogen alone arm *p* =0.001 and in the placebo arm (*p* < 0.001) - Mean PMD increased in estrogen alone users after 12 months follow-up by 0.9% compared to placebo arm; however, the result is not significant (*p* =0.25)
Boyd et al. (2006) [16]	Canada	Case–control	3	- 1748 postmenopausal women - Cases: 365 women who had developed invasive breast cancer at least 12 months after the initial screen. - Matched controls: 879 controls - Age ≥ 50 years	–	PMD	- Age, BMI- Age at menarche - Parity - Number of live births - Age at first birth - Age at menopause - Breast cancer in first-degree relatives (0, 1, 2+)	- Percent density in the baseline mammogram was among cases greater in current users of hormones that in never users (difference = 5.0%, *p* < 0.001), but the difference was smaller and no significant in controls (difference = 1.6%, *p* = 0.3) - Average PMD increased significantly with increasing exposure to HRT among cases, but not in controls
Topal et al. (2006) [34]	Turkey	Cohort	2	113 postmenopausal women - Age ≥ 50 years	- Estrogen alone - CEP - SEP	-BI-RADS	-	- In total 26 women showed MD increase after HRT use. At first mammography, 24 women (92.3%) showed increase in MD and in second mammography, 2 women (7.7%) showed MD - 23 women (38.3%) of CEP, 2 women (12.5%) of SEP, and 1 woman (2.7%) of estrogen alone user showed increase in MD - Increase in MD was more common among women with CEP than other groups of HRT (*p* = 0.0009) - Women were examined according to the progestin dose, in the CEP users 60% of women with higher progestin dose (5mg/day) revealed a MD increase, only 16.7 % of women with lower progestin dose (2.5 mg/day) increase MD. The difference between these two groups were statistically significant (*p* < 0.05)
McTieranan et al (2005) [26]	United States	RCT	1	- 413 postmenopausal women - Age range 50–79 years	CEP	PMD	- Treatment assignment - Mammographic density at baseline and change in density at follow-up - Baseline characteristics (age, body mass index, and race/ethnicity)	- Mean PMD was increased by 6.0% at year 1 in CEP but decreased in never HRT users (*p* < 0.001). After 2 years, the mean changes in PMD increased by 4.9% in CEP group and decreased by 0.8% in never HRT users. - Approximately 75% of the women assigned to CEP group experienced an increase in PMD
Marchesoni et al. (2005) [4]	Italy	RCT	1	- 103 postmenopausal women - Age range 47–56 years - 1- year follow-up study	CEP	Wolfe	–	- After 12 months of HRT 16 out to 35 (45.1%) of CEP users had increased in MD compared to never HRT users and results were highly significant (*p* < 0.001)
Heng et al. (2004) [25]	Singapore	Cross-section		- 29,193 women - Age 45–69 years	–	PMD	- Age - Age at menarche - Menopause - Ever use of OCs or HRT - Smoking - Family history of breast cancer - Height, weight - Parity - Age at first delivery - Menopausal status - History of a breast biopsy	- Use of HRT was associated with higher PMD by 4.4% and duration of using HRT was also significantly associated with higher PMD by 0.07% with *p* = 0.001 in age-adjusted analysis but not in multivariate analysis - HRT use was positively associated with increase dense area in breasts by 3.61 (cm^2^) and duration of HRT was associated with increase the dense area by 0.06 (cm^2^) with *p* = 0.01 in age-adjusted analysis but not in multivariate analysis
Greendale et al. (2003) [31]	United States	RCT	1	- 571 postmenopausal with - Age range 45–64 years	- Estrogen - SEP - CEP	BI-RADS	- Mammographic percent density at baseline - BMI - Daily grams of alcohol consumed - Cigarette smoking - Levels of physical activity - 12-month change in BMI - Randomization and blocking variable (i.e., clinic site and hysterectomy status)	-After 12 months follow-up the absolute mean changes in MPD were observed in CEP and SEP users; 4.76% (95% CI 3.29–6.23%) and 4.58% (95% CI 3.19–5.97%) respectively. No changes in MD was observed in estrogen users compared to never HRT users -A modest 3–5% increase in MPD was observed among women who were treated with combination HRT and those increases did not differ by progestin formulation or schedule
Gapstur et al. (2003)[23]	United States	Cross-section	4	- 296 Hispanic women - Age range ≥ 40 years - Premenopausal women (*n* = 105) - Postmenopausal women (*n* = 191)	–	PMD	–	- PMD was significantly higher for postmenopausal women who currently use HRT compared to never/past users; this difference was 3.3% (*p* = 0.03)
Christodoulakos et al. (2003) [36]	Greece	Cohort	2	- 121 postmenopausal women - Age 38–66 years - 1-year follow-up study	- Estrogen alone - CEP	Wolfe	-	- MD did not increase in never HRT users after 12 months of follow-up. Two women (8%) in estrogen alone group showed an increase in MD - Four women (11.8%) in CEP group showed increase in MD. The results suggested that HRT may suspend breast involution but does not increase MD in majority of women. In the minority of patients who show a density increase, the magnitude of this increase varies according to the regimen used

### Data extraction

Data extraction included the information about author and year of publication, country, study design, quality of study design, sample size and characteristics of the participants, HRT regimens, MD assessment, confounders included in the final analysis, final results and findings are included in Table 1 from each study. The term “progestin” has been used to replace all progesterone synthetic names, such as progestogen and progesterone.

## Results

### Study characteristics

**Fig. 1:**
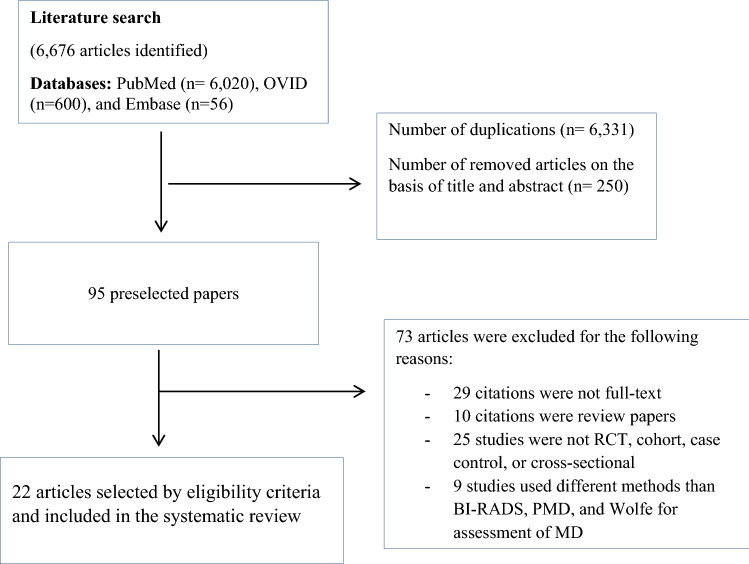
Flow chart of the search and selection process for articles included in the systematic review

The initial search identified 6676 articles. Of these, 6331 were removed due to duplication. Of the remaining 345 articles, 250 were excluded as not relevant based on thorough review of titles and abstracts, and 95 were preselected for further evaluation. Of the 95 remaining articles, 72 did not fulfill the inclusion criteria (Fig. 1). Thus, only 22 articles published between 2002 and 2019 (6 cohort, 6 RCT, 5 case–control and 5 cross-sectional studies) were selected. These 22 studies were conducted in North America (*n* = 12), Europe (*n* = 6), and Asia (*n* = 4) (Table 1).

In all 22 included studies, at least one of the three main methods of MD assessment (PMD, BI-RADS and Wolfe) was used. 12 studies used PMD [16–27], 8 studies used BI-RADS [28–35], and 2 studies assessment MD according to the Wolfe criteria [4, 36].

### State of HRT exposure and MD

#### Ever and never HRT users

From all 22 studies, there were *n* = 12 (54.5%) which investigated the association between ever and never use of HRT and MD [4, 20–22, 26, 27, 31–33, 36]. All these studies reported a significant increase in MD among ever HRT users compare to never users; however, the results from two studies did not reach the statistical significance (Table 1) [22, 25]. In a RCT of 695 postmenopausal women, at 1-year follow-up the PMD change from the baseline was 1.4% for estrogen users and -0.8% for placebo group with a significant increase in MD among women assigned to HRT than women assigned to placebo [20]. In a case–control study, Harvey et al. showed that there is a statistically significant difference in MD among HRT ever and never users groups (*p*-value < 0.001) with a median difference in MD 54% for ever HER users and 31% for never users. They reported that percent fibrous stroma was 7% higher for ever users compared to never users; however, the result was not statistically significant [24]. In another case–control study, Jeon et al. found positive association between HRT and MD. The odds of having dense breasts increased by 2.13; 95% CI (1.09–4.16) for women who used HRT compared to never users [32].

#### Former, current and never HRT users

Five studies examined the association between HRT use and MD among former, current and never HRT users [16, 17, 19, 23, 28]. Three studies showed that MD increased in current HRT users compared to former and never users. Aiello et al. in a cross-sectional study of 39,296 women reported that odds of having high MD increased significantly in women who were the current HRT users (OR 1.91; 1.81–2.00) and former (OR 1.14; 1.08–1.21) as compared to never users [28]. Couto et al. have in a cross-sectional study of 2,424 postmenopausal women found that PMD was highest in current HRT users followed by former and never users with breast densities of 22.6%, 17.7%, and 16.3%, respectively [19]. Additionally, Gapstur et al. in a small cross-sectional study of 296 Hispanic women found that mean PMD was significantly higher in current HRT users than in former and never HRT users: 18.2%, 14%, and 14% (*p-value* = 0.02), respectively [23].

In a case–control study Boyed et al. showed that mean PMD among cases was greater in current HRT users than in never users (difference, 6%; *p-value* < 0.001) and greater in former users than never users (difference, 3.4% *p-value *0.03). Among controls, current use of HRT was associated with a slightly greater mean PMD; however, the results were not statistically significant [17]. In another case control study, Boyd et al. found that among cases, mean PMD was greater in current (25.1%) and former (28.1%) HRT users than in never users (30.7%; *p-value* < 0.001). However, among controls the results were not statistically significant [16].

Finally, a study which examined the effect of HRT on MD among postmenopausal women who previously used HRT and women who never used HRT and that postmenopausal women with history of HRT use had increased odds of having higher MD (OR 2.1; 1.4–3.3) compared to never HRT users [35].

### HRT regimens and MD

Different HRT regimens were addressed in 14 (63.6%) studies [4, 18–22, 26–31, 34, 36]. Five studies compared the effect of estrogen alone and E + P on MD [19, 20, 27, 28, 30], six studies compared the effect of estrogen alone, CEP, and SEP regimens on MD [21, 22, 29, 31, 34, 36]. Finally, three studies assessed the effect of CEP use on MD compared to never HRT use [4, 18, 26] (Table 1).

#### Estrogen alone versus E + P

The results from all five studies showed that E + P users had higher MD compared to estrogen alone users [19, 20, 27, 28, 30]. In a RCT of 695 postmenopausal women, Crandall et al. found that at 1-year follow-up changes in PMD were statistically greater among women assigned to E + P than women assigned to estrogen alone (*p-value* = 0.001) [20]. A cohort study of 1240 postmenopausal women showed that PMD at the first and second mammograms were higher in E + P users than in estrogen alone and never HRT users [27]. Additionally, in a large cross-sectional study of 39,296 postmenopausal women, Aiello et al. reported that current E + P users had significantly increased odds of having dense breasts (OR 1.98; 1.87–2.09) followed by estrogen alone users (OR 1.71, 1.95–1.87) compared to never HRT users [28]. Couto et al. have in a cross-sectional study showed that current E + P users had significantly higher PMD (25.4%) than current estrogen (18.9%) and never users (16.3%) [19]. Finally a small retrospective study of 467 postmenopausal study showed that women using E + P more than 4 years had significantly higher mean MD compared to women who used estrogen only *(p-value* = 0.01); however, after adjustment for other variables the association between MD and HRT regimens diminished [30].

#### Estrogen alone versus CEP and SEP

From six studies which compared the association between estrogen alone, CEP, and SEP regimens on MD [21, 22, 29, 31, 34, 36], four studies showed that CEP users had significantly higher MD compared to SEP and estrogen alone users [29, 31, 34, 36]. In a RCT study of 571 postmenopausal women randomly assigned to receive placebo, daily estrogen alone, SEP, or CEP, after 12 months of follow-up the absolute mean increase in PMD was observed in CEP and SEP users; 4.76% and 4.48%, respectively. No changes in MD were observed in estrogen alone users compared to never HRT users [31]. Additionally, in a retrospective study of 113 of healthy postmenopausal women, Topal et al. showed that 38.3% of CEP users, 12.5% of SEP users and 2.7% of estrogen alone users had increased in MD. Increase in MD was more pronounced among CEP users than other regimens of HRT (*p-value* = 0.009) [34]. Finally, Carmona-Sanchez et al. reported that after 5 years of HRT use, MD increased significantly in 21.8% women receiving SEP versus 38.8% of women receiving CEP (*p-value* = 0.039) [29].

Two studies showed that SEP users had slightly higher MD compared to CEP and estrogen alone users. In a RCT study by Crandall et al. at 12 months, PMD was significantly increased in SEP users by 4.6% and in CEP users by 4.4% [22]. In another RCT by Crandall et al. increase in PMD was 1.2% among estrogen users, 4.7% among CEP users, and 4.9% among SEP users [21].

#### CEP users versus never HRT users

Three studies addressed the increase in MD among CEP users compared to never HRT users. In a RCT study of 103 postmenopausal women, after 12 months 45.1% of CEP users had increased in MD, whereas no changes was observed among never HRT users. The difference between CEP users and never users were statistically significant (*p-value* < 0.001). In another RCT study of 413 postmenopausal women, McTiernan et al. reported an increase in mean PMD by 6% and decrease in MD in placebo group (*p-value* < 0.001) after 1-year follow-up. Finally a case–control study by Byrne et al. showed that women assigned to CEP group had a larger and boarder distribution of MD change (mean change = 9.49%) compared to women in never HRT/placebo group. Women assigned to placebo group experienced decrease in MD over 1 year (mean change = − 0.65%) after adjusting for possible confounders [18].

## Discussion

Our review is the first to systematically investigate the association between HRT and MD, comparing different status of HRT exposure and MD, and finally reporting the effect of different HRT regimens on MD. A consistent finding in the literature is that MD was increased in ever HRT users compared to never users. Furthermore, the highest increase in MD was observed in current HRT users compared to never users [16, 17, 19, 23, 28]; in line with previous studies published before 2003 [37, 38]. Marugg et al. found that, compared to never HRT users, 14.3% of women using HRT showed an increase in MD [37]. Another study concluded that current HRT users were more than twice as likely to have higher MD as never users (OR 2.48; 1.32–4.16) [38].

All selected studies which compared the effect of estrogen alone and E + P on MD showed that E + P users had a higher MD compared to estrogen alone users [19, 20, 27, 28, 30]. Similar to this result, previous studies showed a strong positive association between E + P use and increase in MD [37, 39]. According to Marugg et al. 31% of women treated with E + P showed an increase in MD compared with only 8.7% in the group treated with estrogen alone [37]. Vachon et al. found that odds of having higher MD increased in women using E + P by 1.9 compared to women using estrogen alone [39].

Among the studies which provided data on the sub-type of HRT regimens (CEP, SEP and estrogen alone), four studies reported that women who used CEP, where both estrogen and progestin are taken daily, had higher MD than women who used SEP, where estrogen is used daily but progestin is taken only during a certain time of the month and estrogen alone users [29, 31, 34, 36]. These results are in agreement with the results from other studies that examined the association between HRT and MD with respect to sub-types HRT regimens [40, 41]. In a Swedish study of 31,498 women, Persson et al. has found that MD was significantly increased in 28% of CEP users, 10% of SEP and 5% of estrogen alone users [41]. Another Swedish study by Lundstrom et al. reported that MD was greater among CEP users (52%) than SEP users (13%), estrogen alone users (18%) over 2 years of follow-up [40]. However, two studies showed that SEP users had slightly higher MD compared to CEP and estrogen alone users [21, 22]. It is important to mention that, different HRT regimens influence MD change differently but the mechanism for MD change among CEP and SEP users remain to be unclear. Lundstrom et al. claimed that the inconsistent result on the association between CEP and SEP users with MD maybe due to variation in progestin components, dosage, and duration of administration [40].

The biological explanation in increasing MD with respect to HRT use is not yet fully understood. However, one established hypothesis to explain the increase of MD in relation to use of HRT is based on the breast cell proliferation theory. In menstruating women, it has been observed that breast epithelial cell proliferation is increasing due to high levels of estrogen and progestin [42]. In a cross-sectional study of 56 pre- and 86 postmenopausal women, Hofseth et al. found that, use of HRT especially E + P is associated with higher level of breast epithelial cell proliferation in post- and premenopausal women compared to never HRT users [43]. Breast epithelial cell proliferation is also known as epithelial hyperplasia which defines as abnormal growth and accumulation of cells that line the ducts or the lobules in the breasts [44]. Hofseth et al. also found that breast epithelial cell density was significantly higher in women using HRT, especially in E + P users than estrogen alone and never HRT users [43]. Another hypothesis to explain the association between increasing MD with respect to use of E + P is the stromal oedema theory. Stroma is the major tissue in the breasts therefor any changes in MD primarily reflect alteration of the stroma architecture and composition [44]. Longacre et al. found that stromal oedema is greater in the luteal phase of menstrual cycle which progestin is the main hormone associated with this stage [45]. Therefore receiving E + P should lead to greater MD than estrogen alone.

To our knowledge, this is the first systematic review, which studied not only the effect of different states of HRT exposure on MD but also the effect of different HRT regimens on MD. Strengths of our study are all studies included in this review appropriately reported their study design (RCT, cohort, case–control, and cross-sectional), inclusion criteria, and sampling methods. Sample size was quite large in observational studies and adequate samples were also reported in several RCT studies. Ethical considerations were reported according to the international standards in 18 papers [4, 16–24, 26–28, 31–33, 35, 36]. Most of studies addressed potential confounders, biases and a discussion of limitations. To minimize bias, in this review we used PRISMA checklist and follow chart to ensure transparency and completeness of the reporting (Supplementary material 1). In addition, we assess the quality of each eligible study based on their study designs using the OCEBM hierarchy. Another important strength of this review is that the results can be generalized since the selected studies were from different geographical regions (North America, Europe, and Asia) and these studies presented diverse ethnic groups (Asian, Hispanic, and White). Finally, this review included only peer-reviewed studies, since including unpublished and gray literature increase the risk of publication bias due to the absence of peer-review and low methodological quality. Moreover, the authors did not include their personal opinion or prior knowledge during the review process in order to avoid publication bias.

There are some limitations regarding the studies included in this review. By far the most common limitations mentioned in included studies were; misclassification in the assessment of MD, small sample size, unknown HRT composition, and lack of racial heterogeneity. Other limitations regarding this study are the search for the qualified articles was conducted only in English language, only articles that were accessible electronically were included, and this review found studies with different methodological designs, sample size and demographic factors, therefore due to methodological heterogeneity between studies included in this review it was not possible to conducted a meta-analysis.

## Conclusions

In conclusion, this review showed that MD significantly increased in ever HRT users compared to never users with highest increase in MD among current HRT users. Furthermore, this review found that E + P users had a higher MD compared to estrogen alone users. Results with regards to HRT regimens and MD showed that CEP users had the highest increase in MD followed by SEP users and estrogen alone.

The findings from this systematic review on the association between HRT use and MD can be used in primary prevention of breast cancer incidence as well as secondary prevention of false-negative diagnosis of small tumours. From the primary prevention perspective, our results suggest that the use of HRT should be minimized at the lowest does needed for as short time as possible. In addition, from the secondary prevention perspective, women who are current HRT users and increase in MD is detected by mammography screening may require additional screening procedures, shorter screening intervals, and using advanced imaging techniques such as MRI/ultrasound for detecting small tumours.

### Electronic supplementary material

Below is the link to the electronic supplementary material.

Supplementary file1 (DOCX 15 kb)

## Appendix

An example of the search strategy used in one of the search databases.

## Search strategy for PubMed

**Restriction used:** English Language, year July 2002 to 2019

1. Search (hormone replacement therapy) and mammographic density
2. (Hormone replacement therapy) and breast density
3. (Postmenopausal hormone replacement therapy) and mammographic density
4. (Postmenopausal hormone replacement therapy) and breast density
5. (Estrogen-progestin hormone replacement therapy) and mammographic density
6. (Estrogen-progestin hormone replacement therapy) and breast density
7. (Combined hormone replacement therapy) and mammographic density
8. (Combined hormone replacement therapy) and breast density
9. (HRT) and mammographic density
10. (HRT) and breast density
11. (Estrogen alone hormone replacement therapy) and mammographic density
12. (Estrogen alone hormone replacement therapy) and breast density.

## Abbreviations

BI-RADS: Breast imaging reporting and data system
CEP: Continues estrogen plus progestin
E + P: Estrogen plus progestin
HRT: Hormone replacement therapy
MD: Mammographic density
MWS: Million women study
OCEBM: Oxford center for evidence-based medicine
PMD: Percent mammographic density
PRISMA: Preferred reporting items for systematic reviews and meta-analyses
RCT: Randomized controlled trial
SEP: Sequential estrogen plus progestin
WHI: Women’s health initiative

## References

[1] Jensen PB, Jensen J, Riis BJ, Rodbro P, Strom V, Christiansen C (1987). Climacteric symptoms after oral and percutaneous hormone replacement therapy. Maturitas.

[2] Gallagher JC (2001). Role of estrogens in the management of postmenopausal bone loss. Rheum Dis Clin North Am.

[3] Rossouw JE, Anderson GL, Prentice RL, LaCroix AZ, Kooperberg C, Stefanick ML, Jackson RD, Beresford SA, Howard BV, Johnson KC, Kotchen JM, Ockene J (2002). Risks and benefits of estrogen plus progestin in healthy postmenopausal women: principal results From the Women's Health Initiative randomized controlled trial. JAMA.

[4] Marchesoni D, Driul L, Ianni A, Fabiani G, Della Martina M, Zuiani C, Bazzocchi M (2006). Postmenopausal hormone therapy and mammographic breast density. Maturitas.

[5] Chlebowski RT, Hendrix SL, Langer RD, Stefanick ML, Gass M, Lane D, Rodabough RJ, Gilligan MA, Cyr MG, Thomson CA, Khandekar J, Petrovitch H, McTiernan A (2003). Influence of estrogen plus progestin on breast cancer and mammography in healthy postmenopausal women: the Women's Health Initiative Randomized Trial. JAMA.

[6] Beral V (2003). Breast cancer and hormone-replacement therapy in the Million Women Study. Lancet (London, England).

[7] Ravdin PM, Cronin KA, Howlader N, Berg CD, Chlebowski RT, Feuer EJ, Edwards BK, Berry DA (2007). The decrease in breast-cancer incidence in 2003 in the United States. N Engl J Med.

[8] Boyd NF, Martin LJ, Yaffe MJ, Minkin S (2011). Mammographic density and breast cancer risk: current understanding and future prospects. Breast Cancer Res.

[9] McCormack VA, dos Santos SI (2006). Breast density and parenchymal patterns as markers of breast cancer risk: a meta-analysis. Cancer Epidemiol Biomark Prev.

[10] Hodge R, Hellmann SS, von Euler-Chelpin M, Vejborg I, Andersen ZJ (2014). Comparison of Danish dichotomous and BI-RADS classifications of mammographic density. Acta Radiol Short Rep.

[11] Martin LJ, Minkin S, Boyd NF (2009). Hormone therapy, mammographic density, and breast cancer risk. Maturitas.

[12] Rice MS, Tamimi RM, Bertrand KA, Scott CG, Jensen MR, Norman AD, Visscher DW, Chen YY, Brandt KR, Couch FJ, Shepherd JA, Fan B, Wu FF, Ma L, Collins LC, Cummings SR, Kerlikowske K, Vachon CM (2018). Does mammographic density mediate risk factor associations with breast cancer? An analysis by tumor characteristics. Breast Cancer Res Treat.

[13] World-class systematic review management - A Cochrane technology platform. Covidence (2019). https://www.covidence.org/home/

[14] Moher D, Liberati A, Tetzlaff J, Altman DG (2009). Preferred reporting items for systematic reviews and meta-analyses: the PRISMA statement. BMJ (Clinical Research Ed).

[15] Howick J, Chalmers I, Glasziou P, Greenhalgh T, Heneghan C, Liberati A, Moschetti I, Phillips B, Thornton H. Explanation of the 2011 Oxford Centre for Evidence-Based Medicine (OCEBM) Levels of Evidence (Background Document). Oxford Centre for Evidence-Based Medicine. https://www.cebm.net/index.aspx?o=5653

[16] Boyd NF, Martin LJ, Li Q, Sun L, Chiarelli AM, Hislop G, Yaffe MJ, Minkin S (2006). Mammographic density as a surrogate marker for the effects of hormone therapy on risk of breast cancer. Cancer Epidemiol Biomark Prev.

[17] Boyd NF, Melnichouk O, Martin LJ, Hislop G, Chiarelli AM, Yaffe MJ, Minkin S (2011). Mammographic density, response to hormones, and breast cancer risk. J Clin Oncol.

[18] Byrne C, Ursin G, Martin CF, Peck JD, Cole EB, Zeng D, Kim E, Yaffe MD, Boyd NF, Heiss G, McTiernan A, Chlebowski RT, Lane DS, Manson JE, Wactawski-Wende J, Pisano ED (2017). Mammographic density change with estrogen and progestin therapy and breast cancer risk. J Natl Cancer Inst.

[19] Couto E, Qureshi SA, Hofvind S, Hilsen M, Aase H, Skaane P, Vatten L, Ursin G (2012). Hormone therapy use and mammographic density in postmenopausal Norwegian women. Breast Cancer Res Treat.

[20] Crandall CJ, Aragaki AK, Cauley JA, McTiernan A, Manson JE, Anderson GL, Wactawski-Wende J, Chlebowski RT (2012). Breast tenderness after initiation of conjugated equine estrogens and mammographic density change. Breast Cancer Res Treat.

[21] Crandall CJ, Guan M, Laughlin GA, Ursin GA, Stanczyk FZ, Ingles SA, Barrett-Connor E, Greendale GA (2008). Increases in serum estrone sulfate level are associated with increased mammographic density during menopausal hormone therapy. Cancer Epidemiol Biomark Prev.

[22] Crandall CJ, Karlamangla A, Huang MH, Ursin G, Guan M, Greendale GA (2006). Association of new-onset breast discomfort with an increase in mammographic density during hormone therapy. Arch Intern Med.

[23] Gapstur SM, Lopez P, Colangelo LA, Wolfman J, Van Horn L, Hendrick RE (2003). Associations of breast cancer risk factors with breast density in Hispanic women. Cancer Epidemiol Biomark Prev.

[24] Harvey JA, Santen RJ, Petroni GR, Bovbjerg VE, Smolkin ME, Sheriff FS, Russo J (2008). Histologic changes in the breast with menopausal hormone therapy use: correlation with breast density, estrogen receptor, progesterone receptor, and proliferation indices. Menopause (New York, NY).

[25] Heng D, Gao F, Jong R, Fishell E, Yaffe M, Martin L, Li T, Stone J, Sun L, Hopper J, Boyd NF (2004). Risk factors for breast cancer associated with mammographic features in Singaporean chinese women. Cancer Epidemiol Biomark Prev.

[26] McTiernan A, Martin CF, Peck JD, Aragaki AK, Chlebowski RT, Pisano ED, Wang CY, Brunner RL, Johnson KC, Manson JE, Lewis CE, Kotchen JM, Hulka BS (2005). Estrogen-plus-progestin use and mammographic density in postmenopausal women: Women's Health Initiative randomized trial. J Natl Cancer Inst.

[27] van Duijnhoven FJ, Peeters PH, Warren RM, Bingham SA, van Noord PA, Monninkhof EM, Grobbee DE, van Gils CH (2007). Postmenopausal hormone therapy and changes in mammographic density. J Clin Oncol.

[28] Aiello EJ, Buist DS, White E (2006). Do breast cancer risk factors modify the association between hormone therapy and mammographic breast density? (United States). Cancer Causes Control: CCC.

[29] Carmona-Sanchez E, Cuadros Lopez JL, Cuadros Celorrio AM, Perez-Roncero G, Gonzalez Ramirez AR, Fernandez Alonso AM (2013). Assessment of mammographic density in postmenopausal women during long term hormone replacement therapy. Gynecol Endocrinol.

[30] Chen FP, Cheung YC, Soong YK (2010). Factors that influence changes in mammographic density with postmenopausal hormone therapy. Taiwan J Obstet Gynecol.

[31] Greendale GA, Reboussin BA, Slone S, Wasilauskas C, Pike MC, Ursin G (2003). Postmenopausal hormone therapy and change in mammographic density. J Natl Cancer Inst.

[32] Jeon JH, Kang JH, Kim Y, Lee HY, Choi KS, Jun JK, Oh DK, Lee CY, Ko K, Park EC (2011). Reproductive and hormonal factors associated with fatty or dense breast patterns among Korean women. Cancer Res Treat.

[33] Olsson A, Sartor H, Borgquist S, Zackrisson S, Manjer J (2014). Breast density and mode of detection in relation to breast cancer specific survival: a cohort study. BMC Cancer.

[34] Topal NB, Ayhan S, Topal U, Bilgin T (2006). Effects of hormone replacement therapy regimens on mammographic breast density: the role of progestins. J Obstet Gynaecol Res.

[35] Yaghjyan L, Mahoney MC, Succop P, Wones R, Buckholz J, Pinney SM (2012). Relationship between breast cancer risk factors and mammographic breast density in the Fernald Community Cohort. Br J Cancer.

[36] Christodoulakos GE, Lambrinoudaki IV, Panoulis KP, Vourtsi AD, Vlachos L, Georgiou E, Creatsas GC (2003). The effect of various regimens of hormone replacement therapy on mammographic breast density. Maturitas.

[37] Marugg RC, van der Mooren MJ, Hendriks JH, Rolland R, Ruijs SH (1997). Mammographic changes in postmenopausal women on hormonal replacement therapy. Eur Radiol.

[38] Sala E, Warren R, McCann J, Duffy S, Luben R, Day N (2000). High-risk mammographic parenchymal patterns, hormone replacement therapy and other risk factors: a Case–control study. Int J Epidemiol.

[39] Vachon CM, Sellers TA, Vierkant RA, Wu FF, Brandt KR (2002). Case–control study of increased mammographic breast density response to hormone replacement therapy. Cancer Epidemiol Biomark Prev.

[40] Lundstrom E, Wilczek B, von Palffy Z, Soderqvist G, von Schoultz B (1999). Mammographic breast density during hormone replacement therapy: differences according to treatment. Am J Obstet Gynecol.

[41] Persson I, Thurfjell E, Holmberg L (1997). Effect of estrogen and estrogen-progestin replacement regimens on mammographic breast parenchymal density. J Clin Oncol.

[42] Sendag F, Cosan Terek M, Ozsener S, Oztekin K, Bilgin O, Bilgen I, Memis A (2001). Mammographic density changes during different postmenopausal hormone replacement therapies. Fertil Steril.

[43] Hofseth LJ, Raafat AM, Osuch JR, Pathak DR, Slomski CA, Haslam SZ (1999). Hormone replacement therapy with estrogen or estrogen plus medroxyprogesterone acetate is associated with increased epithelial proliferation in the normal postmenopausal breast. J Clin Endocrinol Metab.

[44] American Cancer Society. Non-cancerous breast conditions. Hyperplasia of the breast (Ductal or Lobular). Version 10.08.2019. https://www.cancer.org/cancer/breast-cancer/non-cancerous-breast-conditions/hyperplasia-of-the-breast-ductal-or-lobular.html. Accessed 20 June 2020

[45] Longacre TA, Bartow SA (1986). A correlative morphologic study of human breast and endometrium in the menstrual cycle. Am J Surg Pathol.

